# Posterior cruciate ligament rupture and all-epiphyseal repair with suture tape augmentation in a 5-year-old girl: a case report and review of the literature

**DOI:** 10.1186/s12887-023-04146-3

**Published:** 2023-06-29

**Authors:** Jinshen He, Kevin Byrne, Jiehui Liang, Anjie Lu, Song Wu

**Affiliations:** 1 Department of Orthopaedic Surgery, the Third Xiangya Hospital of Central South University, Changsha, 410013 Hunan China; 2 Department of Orthopaedic Surgery, University of Pittsburgh Medical Center, Pittsburgh, PA 15213 USA

**Keywords:** Posterior cruciate ligament, Repair, Suture tape augmentation, Case report

## Abstract

**Introduction:**

Only a few case reports regarding pediatric posterior cruciate ligament (PCL) ruptures without bone avulsion exist in the literature. The present study aims to share our experience in the diagnosis, treatment, and prognosis of a child with a proximal PCL tear.

**Materials and methods:**

This article reports a 5-year-old female diagnosed with a proximal PCL tear. The ruptured PCL was repaired with an all-epiphyseal suture tape augmentation (STA) without evidence of growth plate violation.

**Results:**

The suture tape was removed under arthroscopy and revealed the PCL was re-attached at 12 months after the first surgery. And at the time of this report, 36 months after surgery, she was doing well without any problems and with negative posterior drawer test.

**Conclusions:**

Pediatric PCL tear without bone avulsion is rare. However, the torn PCL was noticed healed based on an arthroscopic second-look.

**Supplementary Information:**

The online version contains supplementary material available at 10.1186/s12887-023-04146-3.

## Background

Posterior cruciate ligament (PCL) ruptures are uncommon, largely due to the high relative strength of the fibers [1], and PCL injuries are even more rare in the skeletally immature. There are some studies [2–4] regarding pediatric proximal PCL ruptures without bone avulsion, but the paucity of published data makes clinical decision more difficult and prognostic report especially an arthroscopic second-look rarer. Similar to pediatric ACL injuries, the presence of open epiphyseal growth plates complicates the use of traditional reconstruction techniques. Several recent studies [5–8] in adults reported that suture tape augmentation (STA) may protect and optimize healing in a repaired PCL, eliminating the need to reconstruct a new PCL with an autograft. However, no studies have reported the use of STA in the skeletally immature patients when repairing a ruptured PCL [9]. We present the case of a young child with a proximal PCL rupture that was successfully arthroscopically repaired with STA and without growth plate injury.

## Case presentation

**Fig. 1:**
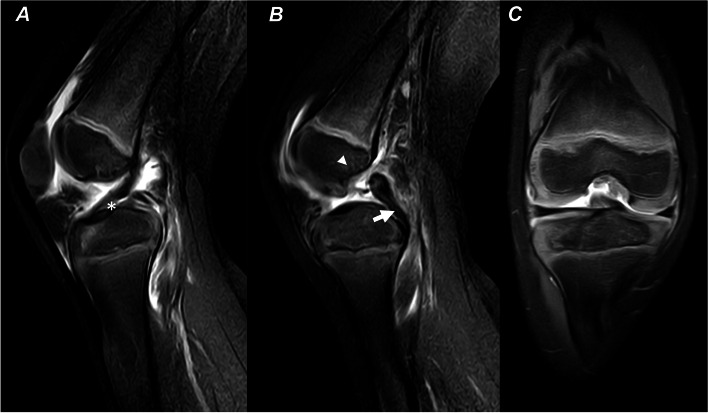
Pre-operation MRI showing an intact ACL (**a**), a complete proximal tear of the PCL (**b**), and intact menisci (**c**). ACL (asterisk); PCL (arrow); Medial condyle (arrowhead)

A 5-year-old girl injured her right knee while playing with her mother. It occurred when the mother hugged the girl’s neck from behind and leaned on the girl’s shoulders with her body weight. The mother’s weight was transferred through the girl’s extended right knee, as she was standing on that one leg. Since this event, the girl complained of right knee pain and the inability to bear weight on the right lower extremity. An adjustable splint was applied in her first visit clinic at the time of the injury and was worn until the patient was seen in our clinic 20 days later. Examination of her knee revealed clinical signs of effusion, no limitation of the range of motion, a positive sagging sign of the right tibia, and a posterior drawer test with over 10 mm displacement (Supplement Video 1). A negative right hip exam was observed. X-Ray did not reveal osseous lesions. Magnetic resonance imaging (MRI) showed a complete proximal tear of the PCL with an intact anterior cruciate ligament (ACL) and intact menisci (Fig. 1). A clinical diagnosis of a grade III PCL tear was made, and the girl was scheduled to be treated with surgery as non-operative management might fail to restore the normal kinematics of the knee and predispose to early degeneration. Written informed consent of the operation was obtained from the patient’s parents.

**Fig. 2:**
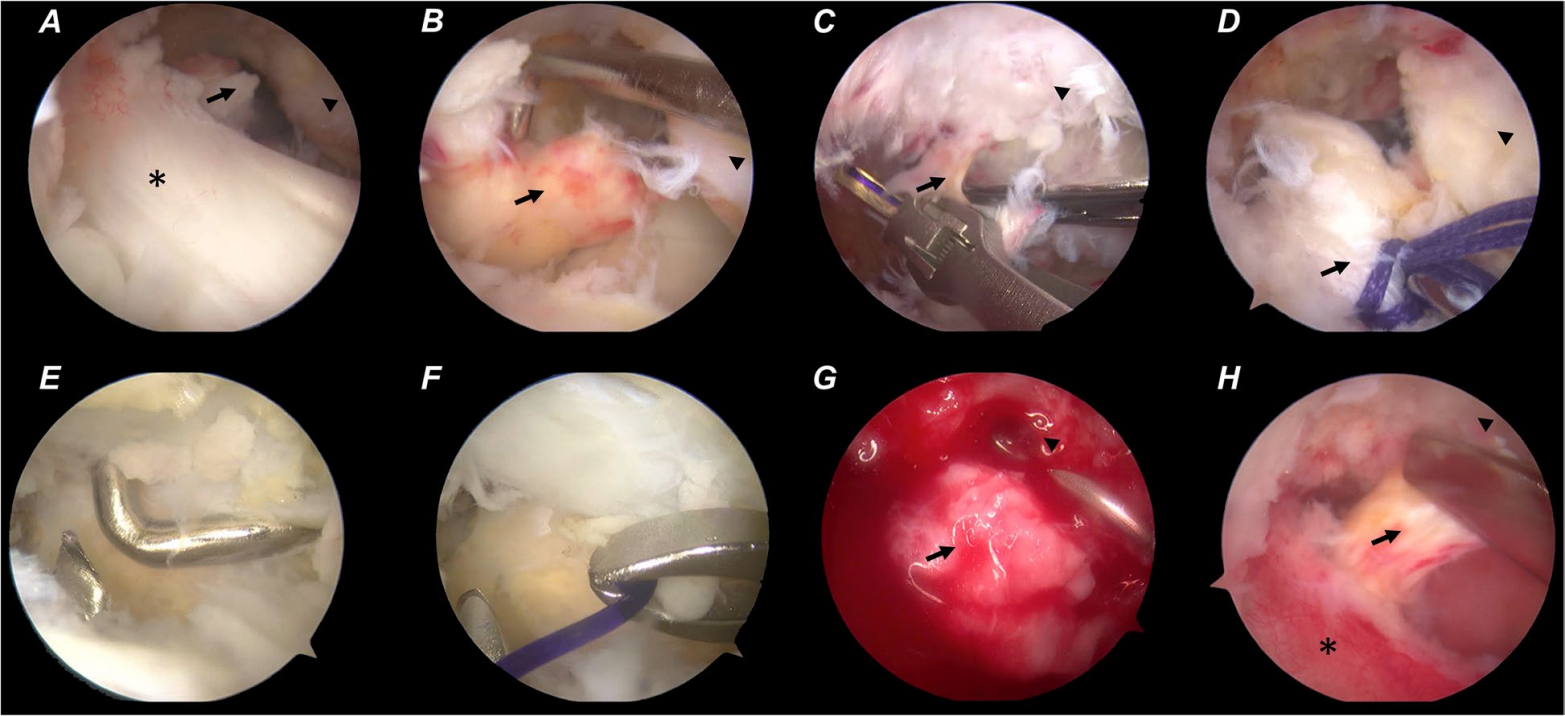
Arthroscopic images of the operation. Only PCL was found ruptured proximally without bone avulsion after general inspection (**a-b**). Absorbable sutures were placed in the PCL stumps by suture passer (**c-d**). STA tibial tunnel was drilled using a 2 mm K-wire (**e–f**). The PRP was injected into the PCL femoral footprint (**g**), and a reduction of the PCL could be seen when the sutures were tensioned in 90° of flexion and anterior drawer (**h**). ACL (asterisk); PCL (arrow); Medial condyle (arrowhead)

**Fig. 3:**
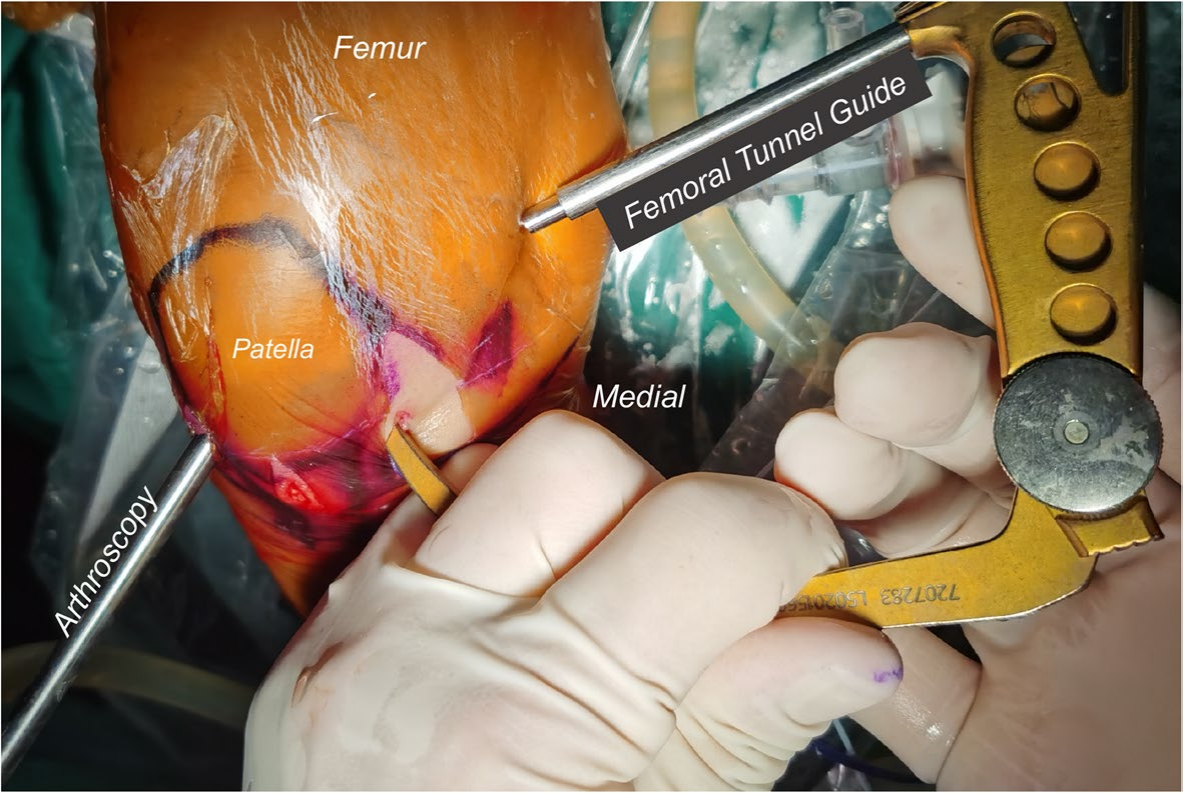
All-epiphyseal femoral tunnel was made via femoral tunnel guide

**Fig. 4:**
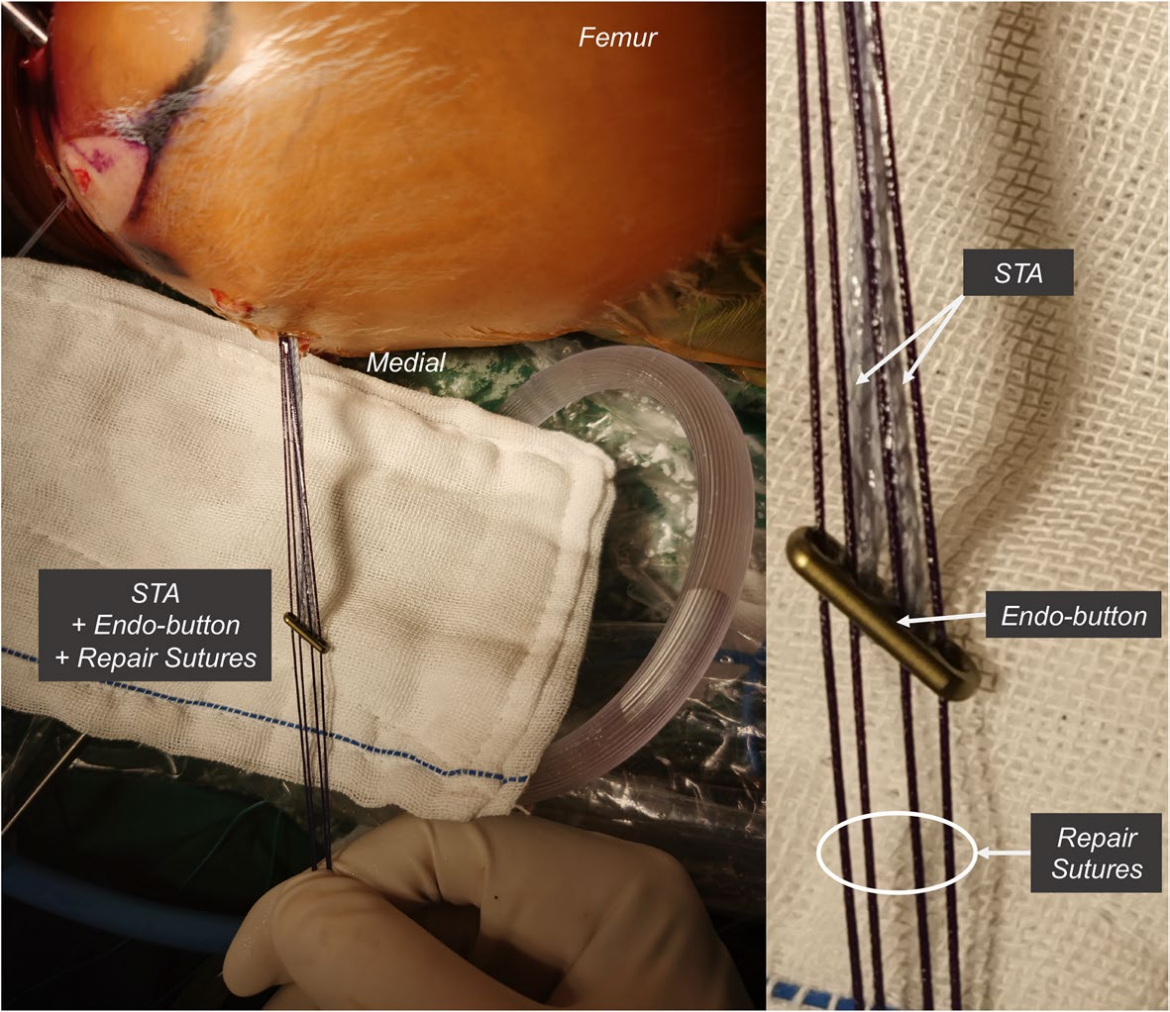
The STA was folded at the eyelets of the cortex-fixation button, and repair sutures were also passed through the eyelets

**Fig. 5:**
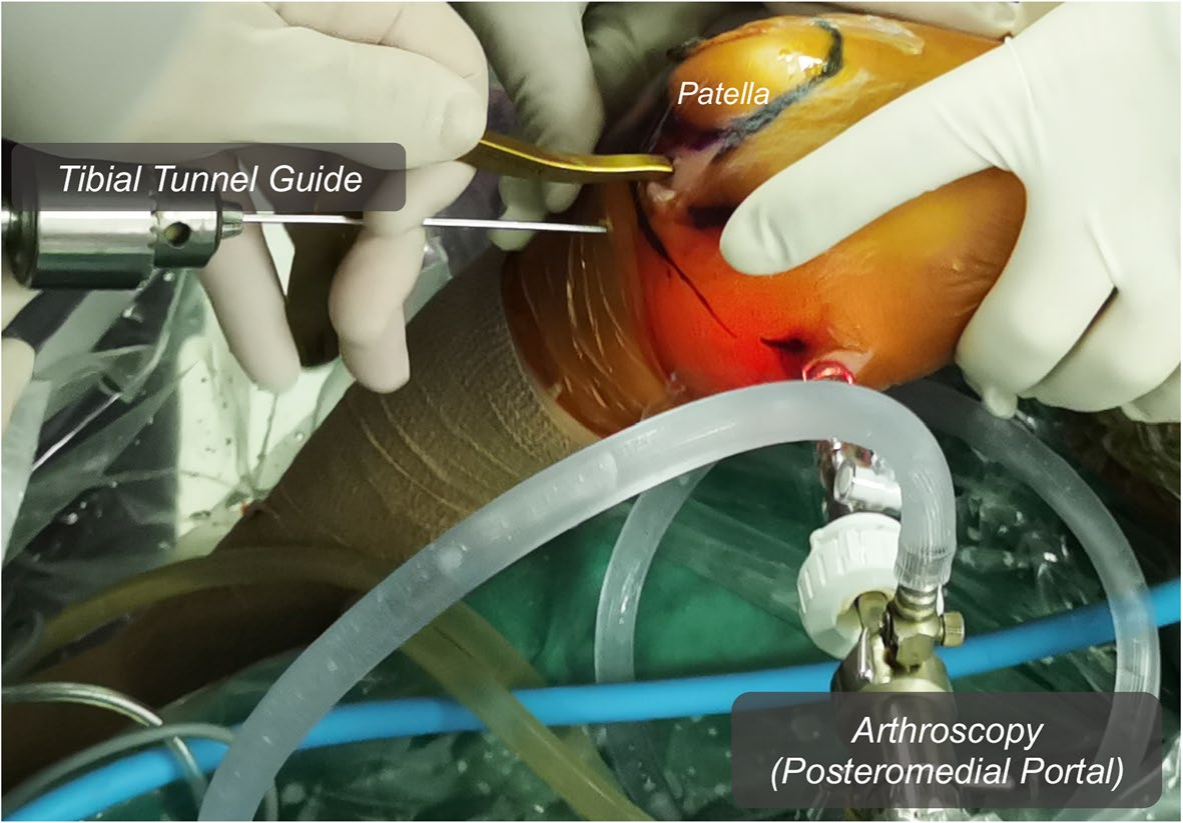
The all-epiphyseal tibial tunnel was made via a tibial tunnel guide

The patient was placed under general anesthesia and positioned supine with a thigh tourniquet and image intensifier set to allow for intra-operative images. Under standard sterile precautions, a standard 30° arthroscope 4 mm in diameter was introduced into the knee for initial assessment. The menisci and ACL were intact. The PCL was found to be ruptured proximally, and without bone avulsion (Fig. 2A-B). Two No. 0 braided absorbable sutures (Coated VICRYL™ Plus, Ethicon) were placed through the tibial PCL stump with a suture passer (Labral scorpion, Arthrex) (Fig. 2C-D). The ends of the sutures were pulled out of the joint through the all-epiphyseal femoral tunnel (Fig. 3), which was drilled using a 2 mm K-wire and a PCL femoral tunnel guide positioned in the PCL femoral footprint (Smith & Nephew) using fluoroscopy. The STA (internal brace, Arthrex) was folded at the eyelets of the cortex-fixation button (Endo-button, Smith & Nephew), and the repair sutures were passed through the eyelets as well (Fig. 4). The end of the STA was extracted out of the joint through an all-epiphyseal tibial tunnel (Fig. 5), which was drilled using a 2 mm K-wire (Fig. 2E-F) and a PCL tibial tunnel guide (Smith & Nephew) manually using fluoroscopy. To tension the PCL, the PCL repair sutures were tied at the surface of the cortex-fixation button on the femur, followed by tibial epiphyseal fixation of the STA. Platelet-rich plasma (PRP) was injected into the PCL femoral footprint (Fig. 2G), and a reduction of the PCL could be seen (Fig. 2H) when the sutures were tensioned in 90° of knee flexion with an anterior drawer force applied to reduce any posterior tibial sag.

**Fig. 6:**
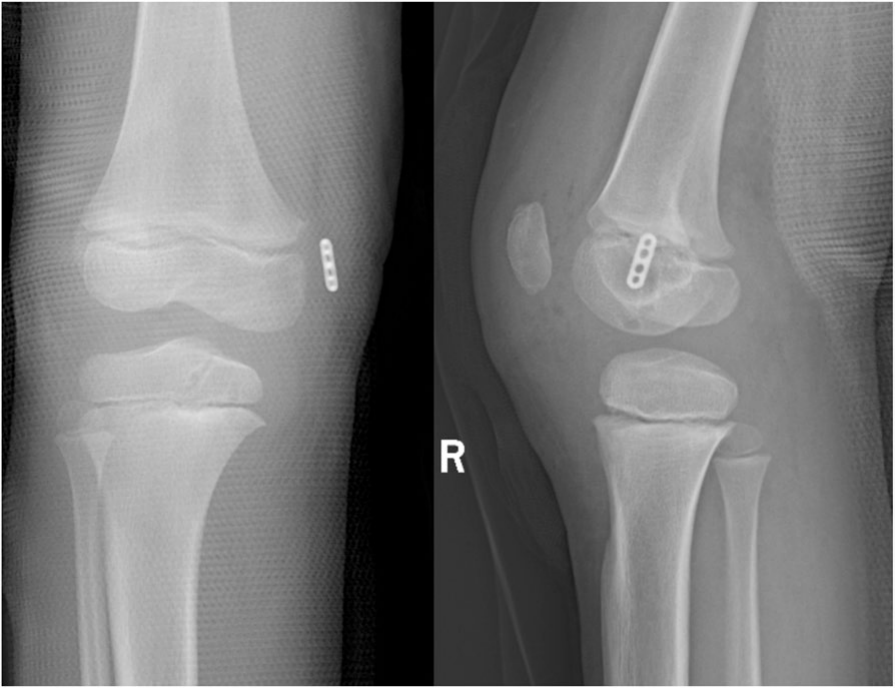
Post-operation X-ray. The cortex-fixation button was placed distal to the growth plate; the gap between the button and cortex is the epiphyseal cartilage

**Fig. 7:**
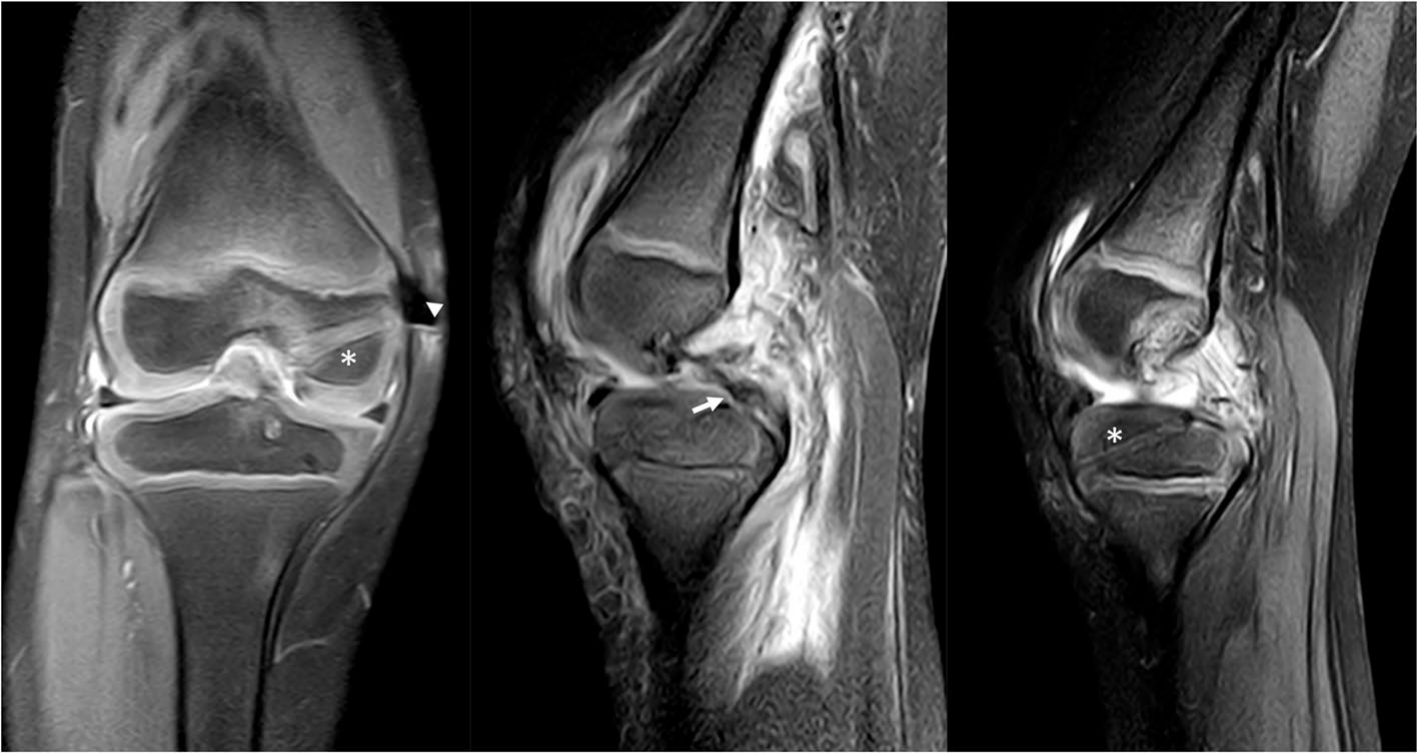
Post-operation MRI. All-epiphyseal bone tunnel (asterisk); Repaired PCL (arrow); Cortex-fixation button (arrowhead)

**Fig. 8:**
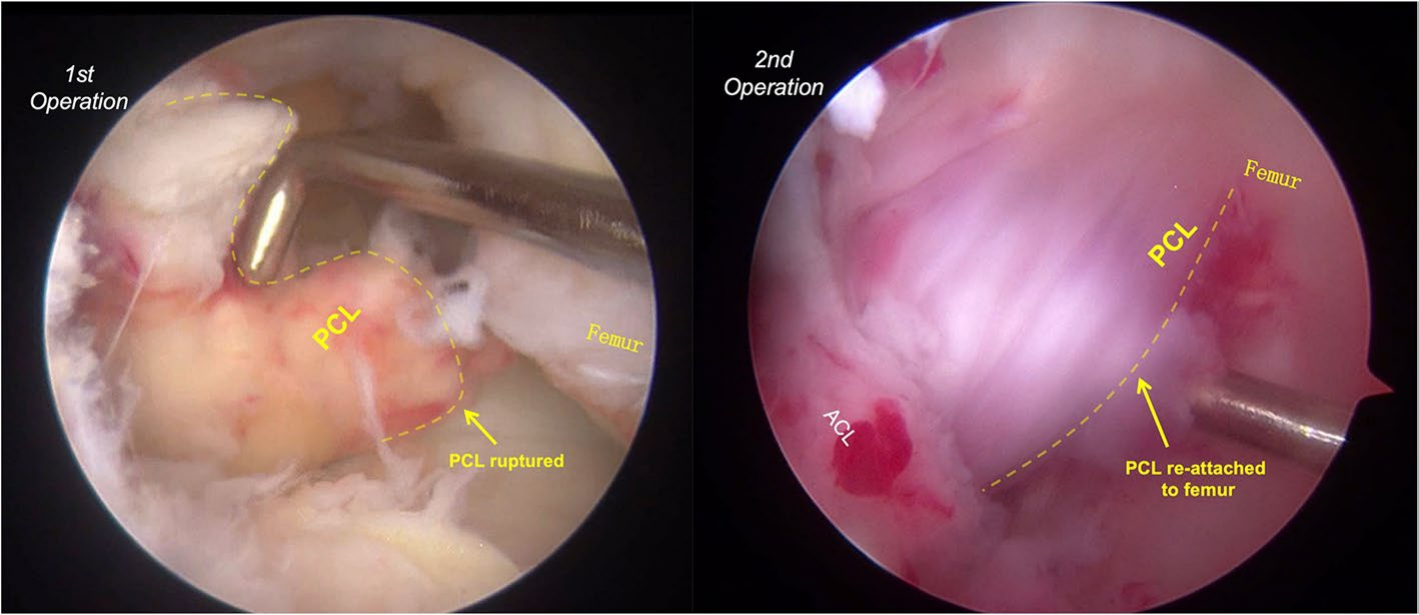
The PCL shape between the first operation (PCL ruptured) and the second operation (PCL healed)

Immediately post operation, the posterior drawer test was negative (Supplement Video 1). The ruptured PCL was repaired with an all-epiphyseal STA without evidence of growth plate violation (Figs. 6 and 7). The child was allowed to walk in an adjustable splint with the help of crutch and was sent to rehabilitation for quadriceps strengthening among the first 4 weeks after the operation. The splint could allow the knee with full range of motion. After 4 weeks post-operation, the crutch and the splint were not applied. At 12 weeks post-operative, she was able ambulate without pain and could take part in all pre-injury activities. (Supplement Video 1) The suture tape was removed under arthroscopy and revealed the PCL was re-attached (Fig. 8) at 12 months after the first surgery. And at the time of this report, 24 months after surgery, she was doing well without any problems and with negative posterior drawer test. Meanwhile, based on the physical examination, no varus or valgus deformity was noticed; and the length of the lower limbs were equal. However, the longer follow up result about the limbs related to growth plate is still unknown, which would be evaluated by annual clinical assessment.

## Discussion and conclusion

To the best of our knowledge, this is the first reported case utilizing an all-epiphyseal repair with STA in a skeletally immature patient with a proximal PCL tear. In the present case, the mechanism of injury was hyperextension. This caused failure at the femoral attachment since the femoral chondro-osseous junction is the weakest point in children’s PCLs [1, 10]. A delay of more than 2–3 weeks after injury could result in a time-dependent decrease in tissue quality [11]. Therefore, primary repair combined with STA augmentation was applied in the present case.

The literature on the treatment of pediatric PCL lesions without bone avulsion (Table 1) is sparse. The youngest case report of arthroscopic PCL repair was reported by Lobenhoffer et al. [2], who reported encouraging results with a normal range-of-motion and firm anterior–posterior endpoint in a 3-year-old boy. Nevertheless, MacDonald et al. [12] reported anterior knee pain with nonoperative treatment in a 5-year-old girl. Moreover, a study of seven children [13] with femoral avulsion fractures of the PCL treated conservatively or surgically found that non-operatively treated children had poor functional results. Considering the lack of knowledge regarding the natural history of PCL injuries in children, the most logical strategy is to follow the protocol of adult PCL treatment, which may include repair of the PCL stump securely and without the injury of the growth plate.

**Table 1:** Pediatric PCL ruptures without bone avulsion

Study	*Journal*	*Year*	*Country*	*Cases*	*Tear Location*	*Treatment*
***The present study***		2021	China	5-year-old girl	Proximal	Primary repair with STA
Lobenhoffer [2]	*Arthroscopy*	1997	Germany	3-year-old boy	Proximal	Repair by trans-osseous absorbable sutures
Wheatley [3]	*Arthroscopy*	2002	USA	15-year-old girl	Proximal	Repair by trans-osseous nonabsorbable sutures
MacDonald [12]	AJSM	2003	Canada	6-year-old boy	Midsubstance	Nonoperative
Scott [14]	BMJ Case Rep	2011	UK	4-year-old boy	Midsubstance	Nonoperative
Pisanu [4]	Arth Tech	2019	France	Age not report	Proximal	Repair by trans-osseous nonabsorbable sutures

Treatment of pediatric PCL injuries always poses a dilemma [14]. One main reason to consider arthroscopic PCL repair over reconstruction includes the potential preservation of the native fibers [15]. Additional benefits include the smaller diameter of the bone tunnels drilled and the absence of graft harvesting [16–18]. The decision to repair PCL is made intraoperatively upon verification that the remnant can be reapproximated to the femoral footprint. For that reason, an alternative plan, such as physis-sparing hamstring graft PCL reconstruction [19, 20], should always be presented and discussed with the child and parents. Furthermore, long-term data and consistent clinical evaluation are lacking for this specific technique. These issues must also be addressed during preoperative consultation regarding the risks and benefits of the procedure. Meanwhile, quadriceps strengthening during recovery and rehabilitation remains crucial in this case [21].

The present case featured somewhat unusual characteristics. First, the patient came to our clinic almost three weeks after the injury. Second, the parent refused any procedures that violated the growth plate. The present technique avoided the growth plate entirely and allowed for repair of the PCL in combination with STA [22–24] in the event of decreased tissue quality. Additionally, to potentially promote healing of the PCL at the site of the injury, PRP was applied to the femoral footprint [25]. However, the application of PRP is still controversial, especially in the graft healing. It was not recommended to routinely applied in all ligament surgeries. The updated systematic review [26] indicated reduced postoperative pain and improved knee function in the short and medium terms after the PRP injection, however, the long term is ineffective. PRP does not accelerate the healing of grafts. More studies would be required in the future.

In conclusion, proximal PCL tears in skeletally immature patients are rare. This is the first report of a pediatric PCL repair with STA using the physis-sparing technique as a possible method to restore knee function and stability.

## Supplementary Information

**Additional file 1: Supplement Video 1.** Captured steps of PCL repair.

## Data Availability

The datasets are available from the corresponding author on reasonable request.

## Abbreviations

PCL: Posterior cruciate ligament
CT: Computed tomography
MRI: Magnetic resonance imaging
ACL: Anterior cruciate ligament
PRP: Platelet-rich plasma
STA: Suture tape augmentation
